# The role of healthy personality, psychological flexibility, and coping mechanisms in university students’ mental health in China

**DOI:** 10.3389/fpsyg.2025.1578793

**Published:** 2025-08-01

**Authors:** Xiao Yang, Kartini Ilias, Khairil Anuar Md Isa, Qing-hong Li, Hai-bin Wang, Huan Li

**Affiliations:** 1 Faculty of Health Sciences, Universiti Teknologi MARA, UiTM Puncak Alam Campus, Puncak Alam, Selangor, Malaysia; 2 School of Educational Sciences, Huangshan University, ShuaiShui Campus, Huangshan, Anhui, China; 3 Business and Consumer Health Sciences (BiZ-HeALtH), Universiti Teknologi MARA, Cawangan Selangor, Puncak Alam Campus, Selangor, Malaysia; 4 School of Mathematics and Statistics, Huangshan University, ShuaiShui Campus, Huangshan, Anhui, China; 5 School of Economics and Management, Huangshan University, ShuaiShui Campus, Huangshan, Anhui, China

**Keywords:** personality health, psychological flexibility, coping mechanisms, mental health, university students

## Abstract

**Background:**

Mental health issues such as anxiety and depression have become increasingly common among Chinese university students, especially after the COVID-19 pandemic. This study explores the relationships between personality health, psychological flexibility, coping styles, and mental health in this population.

**Methods:**

A cross-sectional survey was conducted with 2,528 university students (aged 18–25, 53.26% male). Personality was assessed using the MMPI, psychological flexibility via the AAQ and CFQ-F, and coping styles using the SCSQ. Mental health outcomes were gathered as part of a university-wide assessment.

**Results:**

After controlling for demographic variables, personality health, psychological flexibility, and coping styles were significantly associated with mental health. Psychological flexibility showed a moderate correlation (*r* = 0.454–0.660, *p* < 0.001). The final multivariate model, including psychological flexibility and coping styles, explained a moderate portion of variance in mental health (Adjusted *R*^2^ = 0.4892, *p* < 0.001).

**Conclusion:**

Psychological flexibility and adaptive coping strategies play a key role in promoting mental health among university students. Targeted interventions in these areas—such as integrating them into university curricula and support programs—can help mitigate anxiety and depression. Though based on a Chinese sample, the findings align with global research, highlighting the cross-cultural relevance of psychological flexibility in mental health interventions.

## 1 Introduction

In the aftermath of the COVID-19 pandemic, the mental health of Chinese university students has exhibited a marked decline (Ibrahim and Alexcius, 2023; Chang et al., 2020; Blake et al., 2009). Incidences of anxiety, depression, and even suicide have become increasingly prevalent on university campuses (Chen et al., 2024; Hu et al., 2023; Guo et al., 2023). According to data from the Chinese Centre for Disease Control, between 16 and 25.4% of students are affected by mental health disorders. Broader studies report that 30–50% of Chinese university students experience mental health difficulties (Han et al., 2020; Guo et al., 2021). At Huangshan University, for instance, 37.8% of students were reported to have psychological issues in the 2022 school-wide Mental Health Test Report (Yang and Han, 2022). Other surveys have revealed that 34.6% of university students show symptoms of depression, and 41.1% experience anxiety (Huang et al., 2022; Fu et al., 2021).

Among the emerging approaches to address these problems, Acceptance and Commitment Therapy (ACT)—a third-wave cognitive behavioral therapy—has gained prominence for its core focus on psychological flexibility and its demonstrated efficacy in promoting mental health during the pandemic (Hu and Chen, 2020; Hayward et al., 2013). It is a central concept that enhances psychological flexibility and can improve Chinese university students’ mood and quality of life (Hussey and Barnes-Holmes, 2012; Jin, 2013). Moreover, it has proven effective in addressing behavioral and psychological issues (Landi et al., 2022), including anxiety and depression among adolescents (Chen et al., 2017). However, while ACT has been validated in various settings, the mechanisms through which psychological flexibility interacts with other psychological constructs—such as personality traits and coping styles—remain insufficiently understood in the context of Chinese university students (Tanhan et al., 2020). A deeper theoretical understanding of these relationships is essential for the effective application of ACT in this population (Li et al., 2015).

According to the Chinese Dictionary of Psychology, mental health is characterized not only by the absence of psychological disorders but also by a well-functioning state of self-awareness, adaptability to one’s environment, and continuous psychological growth (Lin et al., 2003; Lin et al., 2018). Mental health, therefore, involves proactive engagement with life challenges and internal regulation (Li, 2003; Meléndez et al., 2020). A healthy personality serves as the foundation for mental resilience, shaping how individuals perceive themselves and respond to stress (Yıldırım, 2023; Yıldırım and Özaslan, 2022; Mir et al., 2023). Research has shown that specific personality traits are closely associated with mental health conditions such as depression (Fournier et al., 2017; Li et al., 2019), and these associations are further influenced by coping styles (Tee et al., 2022). While positive coping strategies generally foster well-being (Pan et al., 2021), negative coping strategies often have complex and potentially harmful effects (Yuan et al., 2023).

The construct of psychological flexibility, introduced by Hayes et al. (1999), addresses fundamental cognitive and behavioral patterns underlying many mental health issues (Shi et al., 2018). It refers to the ability to remain aware, open, and engaged in the present moment while adapting behavior in alignment with one’s values—even in the presence of negative thoughts or emotions (Hayes, 2019). Two core components of this construct—cognitive fusion and experiential avoidance—have been shown to strongly correlate with mental health symptoms (Twohig et al., 2018), including anxiety and depression, particularly among university students (Yang et al., 2019; Cookson et al., 2020; Lei et al., 2019; Wei, 2014).

Despite a growing body of literature, most studies in China have examined university students’ mental health from an interventionist or symptom-focused perspective. This often overlooks the psychological processes and structural factors—such as personality traits, coping mechanisms, and psychological flexibility—that contribute to the development and persistence of mental health issues. While the relationships between some of these variables have been individually examined, few studies have proposed integrated models or predictive frameworks.

**Figure 1:**
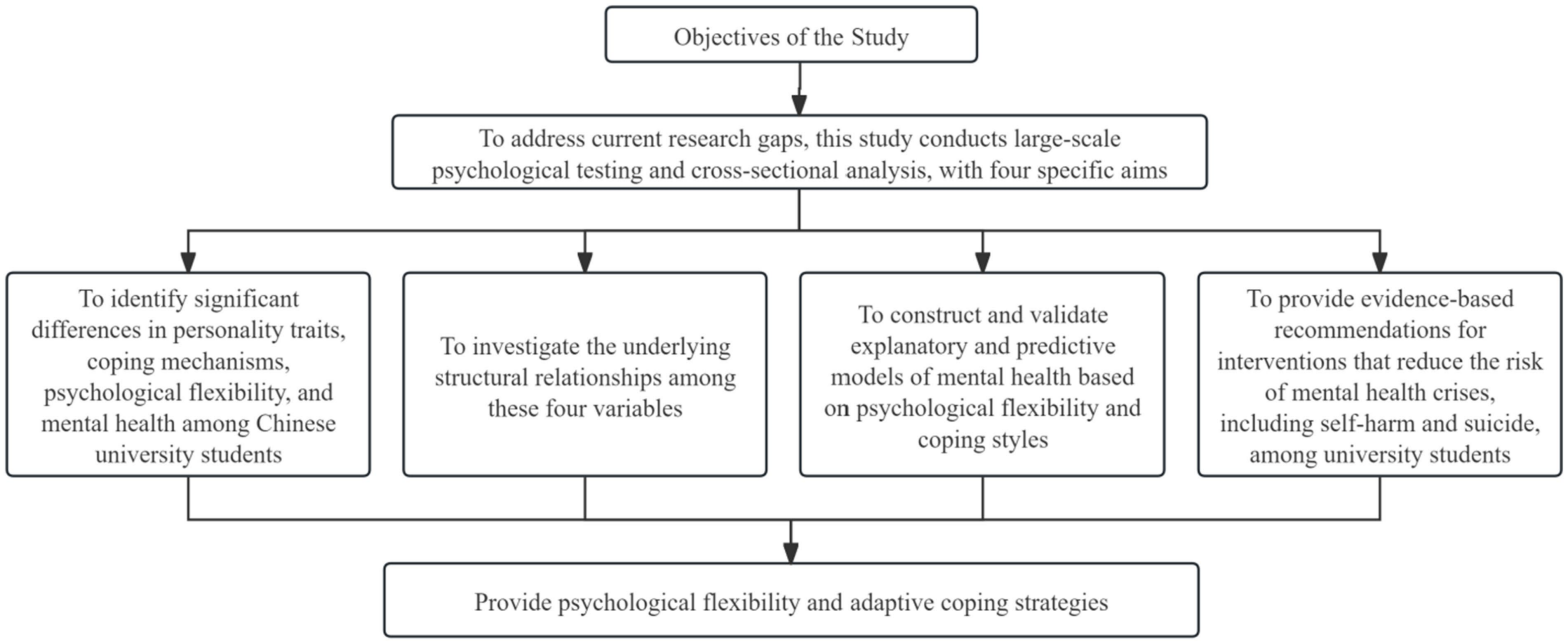
Integrated theoretical model.

This study proposes an integrated theoretical model in which personality traits influence both coping mechanisms and psychological flexibility (Figure 1), and these two factors in turn directly impact mental health. Furthermore, it is hypothesized that psychological flexibility mediates the relationship between personality and mental health, while coping mechanisms may moderate this effect. This conceptual framework allows for the identification of both direct and indirect pathways to psychological well-being, emphasizing process-oriented variables rather than symptom-based outcomes.

Our survey included five categories of variables: sociodemographic characteristics, healthy personality, psychological flexibility, coping mechanisms, and mental health outcomes. Sociodemographic variables included gender (male or female), age (18–25 years), grade (2021, 2022, 2023, or 2024), academic discipline (literature, science, engineering, art, or physical education), and first degree (undergraduate or university level). Healthy personality was measured using the Chinese version of the Minnesota Multiphasic Personality Inventory (MMPI), which consists of 399 items that assess personality-related psychopathology across four validity scales and ten clinical scales. A healthy personality was operationalized as the relative absence of elevated clinical scores, with scores below 60 on each clinical scale indicating non-clinical functioning (Zhou and Zhang, 2020). The clinical scales included Hs (Hypochondriasis), D (Depression), Hy (Hysteria), Pd (Psychopathic Deviate), Mf (Masculinity/Femininity), Pa (Paranoia), Pt (Psychasthenia), Sc (Schizophrenia), Ma (Hypomania), and Si (Social Introversion). The validity scales were Q (Question Scale), L (Lie Scale), F (Infrequency Scale), and K (Correction Scale). Psychological flexibility was assessed using the Acceptance and Action Questionnaire (AAQ) and the Cognitive Fusion Questionnaire (CFQ). Both instruments are widely used in Chinese populations to measure experiential avoidance and cognitive fusion, respectively, with higher scores indicating lower psychological flexibility (Yıldız, 2020). Coping mechanisms were evaluated using the Simplified Coping Style Questionnaire (SCSQ), which includes two subscales measuring positive coping (PC) and negative coping (NC) styles. The SCSQ (Simplified Coping Style Questionnaire) has demonstrated high reliability and cultural validity in Chinese samples. Mental health outcomes were measured using the Symptom Checklist (SCL), a widely used instrument that assesses psychological symptoms across ten dimensions, including SOM (Somatization), O-C (Obsessive-Compulsive), INT (Interpersonal Sensitivity), DEP (Depression), ANX (Anxiety), HOS (Hostility), PHOB (Phobic Anxiety), PAR (Paranoid Ideation), PSY (Psychoticism), and the Global Severity Index (GSI). Additional indicators include the total score (T), the average score (AT), and the number of positive items (NP) (Jin et al., 1984). All instruments were adapted and validated for use in Chinese university populations to ensure cultural appropriateness and psychometric reliability. By emphasizing psychological processes rather than merely symptomatic expression, this study aims to provide a deeper understanding of how psychological flexibility and adaptive coping strategies—supported by stable personality traits—serve as protective factors for the mental health of Chinese university students.

The selection of these variables is grounded in well-established psychological models, including the cognitive-behavioral framework of psychological flexibility (Hayes, 2019; Wu et al., 2019; Xu et al., 2018), and the understanding of coping mechanisms as dynamic factors that shape mental health outcomes (Wang, 2015; Xu, 2022). By examining these variables in combination, the study offers a comprehensive framework for understanding the role of psychological flexibility and coping styles in mental health. This integrated perspective not only fills a critical gap in regional research but also contributes to broader global discussions on student mental health in higher education settings.

## 2 Methodology

### 2.1 Research design

This study is a cross-sectional investigation based on a large sample of observations. The research participants were university students from Huangshan University in Anhui Province, China. Through a school-wide mental health assessment, we examined the relationships between healthy personality, psychological flexibility, coping mechanisms, and mental health. The study was approved by the ethics committees of Universiti Teknologi MARA and Huangshan University and was conducted with the support of the Student Affairs Office and the University Student Mental Health Education Center of Huangshan University.

### 2.2 Research setting

Up to 35% of students at Huangshan University are currently experiencing mental health problems as a result of the COVID-19 pandemic. Unfortunately, incidents of student suicide have occurred, drawing serious concern from relevant administrative departments and highlighting the urgent need to improve mental health support for students. A self-administered demographic questionnaire and a survey consisting of psychological assessments—covering psychological flexibility, mental health, healthy personality, and coping mechanisms—will be distributed to research participants. By analyzing the differences in students’ responses, we aim to identify significant variations across key psychological constructs.

### 2.3 Sampling procedure

To ensure scientific rigor and representativeness, a systematic random sampling strategy was employed. Participants were selected from the full-time undergraduate student population at Huangshan University using a structured sampling interval, ensuring proportional representation across the institution’s various academic departments and academic year levels. This method allowed each individual in the population to have an equal and independent chance of selection, thereby reducing sampling bias and enhancing the generalizability of the findings.

Huangshan University, located in Anhui Province, recruits students from a broad range of regions across mainland China. These include eastern coastal provinces such as Jiangsu and Zhejiang, central provinces like Hubei and Henan, as well as southwestern and northern areas. Consequently, the sample indirectly captures a wide range of geographic, cultural, and educational backgrounds, contributing to regional diversity within the student cohort.

Efforts were made to maintain balance across key sociodemographic variables, including gender, academic year, academic discipline, and first-degree status. Although detailed socioeconomic information was not directly collected, the student body generally reflects middle- and lower-middle-income populations in China, as inferred from institutional enrollment profiles and tuition structures. This diversity ensures that the study’s findings are demographically and contextually relevant to the broader population of Chinese university students.

### 2.4 Measurement tools

This study utilized several validated instruments to assess personality health, psychological flexibility, coping mechanisms, and mental health outcomes, all of which have been culturally adapted and psychometrically verified for use within the Chinese context. A detailed description of each tool and its adaptation process is provided below:

#### 2.4.1 Minnesota multiphasic personality inventory (MMPI, Chinese version)

The MMPI, developed by Professor Song Weizhen’s team at the Institute of Psychology, Chinese Academy of Sciences, is a widely adopted instrument in China for assessing personality health. The scale consists of 399 items and includes four validity scales (Q, L, F, K) and ten clinical scales (Hs, D, Hy, Pd, Mf, Pa, Pt, Sc, Ma, Si). It is an adaptation of the original American MMPI, with modifications to enhance cultural relevance and linguistic appropriateness. The Chinese version has demonstrated strong psychometric properties, including test–retest reliability coefficients ranging from 0.70 to 0.90, and is extensively used in both clinical and occupational settings in China.

#### 2.4.2 Acceptance and action questionnaire (AAQ)

The AAQ measures psychological flexibility by assessing individuals’ tendencies toward experiential avoidance and cognitive fusion. Higher scores on the AAQ indicate lower levels of psychological flexibility. This instrument has been adapted for Chinese university students and exhibits strong internal consistency (Cronbach’s *α* = 0.800) and acceptable test–retest reliability (α = 0.883) in Chinese samples.

#### 2.4.3 Cognitive fusion questionnaire (CFQ)

The CFQ is designed to assess cognitive fusion, one of the core components of psychological inflexibility. The Chinese version has been validated for non-clinical populations and demonstrates high internal consistency (α = 0.86) and test–retest reliability (*r* = 0.82).

#### 2.4.4 Simplified coping style questionnaire (SCSQ)

Developed by experts at the Shanghai Mental Health Centre, the SCSQ is specifically designed for the Chinese population. It consists of two subscales: Positive Coping (PC) and Negative Coping (NC). The scale captures culturally relevant coping behaviors and has been widely employed in Chinese psychological research. The SCSQ has demonstrated excellent internal consistency, with an overall Cronbach’s α of 0.90.

#### 2.4.5 Symptom checklist (SCL)

The SCL is a comprehensive self-report inventory used to evaluate a broad range of psychological symptoms, including anxiety, depression, and somatization. The Chinese version was translated and validated by a team at the Shanghai Mental Health Centre and has shown robust reliability across multiple studies. Internal consistency coefficients for its subscales range from 0.75 to 0.97, making it one of the most commonly used instruments for mental health assessment in China (Jin et al., 1984).

## 3 Participants and procedure

### 3.1 Participants

The study sample consisted of 2,528 students from Huangshan University, selected from a total student population of approximately 20,000. Participants ranged in age from 18 to 24 years, with a mean age of 20.5 years (SD = 1.5). Gender distribution in the sample included 1,345 male students (53.26%) and 1,183 female students (46.74%). The sample was evenly distributed across four academic cohorts: 25.4% from the 2020 cohort, 26.3% from 2021, 24.8% from 2022, and 23.5% from 2023. Regarding academic disciplines, 18.4% of participants were enrolled in literature and history, 22.1% in science, 27.8% in engineering, 13.2% in physical education, and 18.5% in art. In terms of degree status, 91.8% were pursuing undergraduate degrees, while 8.2% had completed university-level education.

The sample was obtained using a systematic random sampling method. In April 2024, a school-wide mental health assessment was administered to all enrolled students via the university’s official online mental health management system. The assessment was completed voluntarily, and the system automatically calculated and stored standardized scores for each scale in Excel format. After excluding 212 incomplete or invalid submissions, a total of 20,641 valid responses were retained.

### 3.2 Procedure

A comprehensive list was created linking student ID numbers to their psychological assessment results. Systematic sampling was then applied by selecting every 8th student from the list, based on the formula *k* = N/n, where *N* = 20,641 and *n* = 2,528. A random number generator was used to determine the initial selection point. Additionally, the MMPI includes built-in validity checks, and 52 responses were excluded for failing to meet these criteria. The final analytic sample consisted of 2,528 valid participants.

Before participation, all students received detailed information about the study’s purpose and procedures via pop-up windows within the online system and were required to provide informed consent prior to beginning the psychological assessments. All procedures adhered to ethical guidelines, and the confidentiality and voluntary nature of participation were strictly maintained.

### 3.3 Statistical analyses

All analyses were conducted using the packages gtsummary, tidyverse, polycor, dplyr, corrplot, tools of R Studio for Windows (R version 4.3.32024-02-29 ucrt).

First, descriptive statistical analyses were conducted to examine the central tendency and dispersion of the dataset. Histograms and the Kolmogorov–Smirnov test were used to assess the normality of continuous variables, while boxplots were applied to identify outliers, missing values, and possible anomalies. Frequencies and percentages were reported for categorical variables, and means and standard deviations were reported for continuous variables. Continuous variables were further classified by gender, grade, academic subject, age, and first degree.

Second, independent-samples t-tests were conducted to examine whether significant differences in continuous variables existed based on gender and first degree. Prior to the tests, the normality of the relevant continuous variables was verified, and Levene’s test was used to assess the homogeneity of variances. Mean values and standard deviations were presented for each group.

Third, one-way analysis of variance (ANOVA) was used to examine whether continuous variables significantly differed by grade, age, and academic subject. Levene’s test was again applied to assess the equality of variances. Results were reported using means and standard deviations. The assumption of homogeneity of variance was verified through Levene’s test.

Next, Pearson correlation analysis was conducted to explore the strength and direction of the relationships between pairs of continuous variables. Prior to correlation analysis, the normality of the dependent variables was assessed. Control variables with statistically significant effects—such as gender, grade, and academic subject—were included to adjust for confounding effects. Personality health was measured using the MMPI (scores from each factor), psychological flexibility using the AAQ and CFQ total scores, coping styles using SCSQ positive and negative scores, and mental health using the SCL factor and total scores.

Finally, multiple linear regression analyses were conducted on variables significantly correlated with mental health (measured by the total score of the SCL). A hierarchical regression approach was used to evaluate the incremental predictive value of each variable group. Model 1 included only personality health; Model 2 added psychological flexibility; Model 3 included coping styles; and Model 4 combined all variables. This stepwise strategy allowed us to assess how the inclusion of each factor improved the explanatory power of the model, while simultaneously controlling for gender, age, and academic subject.

Interaction terms were introduced by multiplying key independent variables to explore whether any moderating effects existed—particularly whether psychological flexibility moderated the effects of personality traits or coping styles on mental health. The variance inflation factor (VIF) was used to assess multicollinearity, with a VIF value above 10 indicating potential statistical instability. Model fit and assumptions were verified by inspecting scatterplots of unstandardized predicted values and residuals, ensuring the assumption of homoscedasticity was met. Histograms of the residuals were plotted to assess the normality of the error distribution. When all assumptions were satisfied, adjusted regression coefficients (*β*), 95% confidence intervals, associated *p*-values, and significance levels were reported.

Given the multidimensional and interrelated nature of the constructs examined in this study—namely, personality health, psychological flexibility, coping mechanisms, and mental health outcomes—it was both theoretically and statistically appropriate to explore potential mediation and moderation effects. The results of the correlation and regression analyses suggest that while personality health is modestly associated with mental health outcomes, its predictive power is substantially enhanced when psychological flexibility and coping mechanisms are incorporated. This supports the hypothesis that psychological flexibility may mediate the relationship between personality and mental health—indicating that individuals with healthier personality traits may exhibit greater psychological flexibility, thereby improving their mental well-being. Furthermore, due to individual differences in these relationships, psychological flexibility may also function as a moderator that buffers or amplifies the effects of personality traits and coping styles on psychological distress.

## 4 Results

We obtained 2,528 valid psychological test results between April 2 and April 12, 2024, through the mental health management system for university students at Huangshan University. Since Chinese university students are required to participate in annual mental health assessments, the dataset initially had minimal missing data. Participants were aged between 18 and 25 years and represented four academic cohorts (2020 to 2023) and five academic disciplines: literature and history, science, engineering, physical education, and art. The gender distribution was 1,345 male students (53.26%) and 1,183 female students (46.74%).

Table 1 presents the sociodemographic characteristics of the study sample. The male-to-female ratio is approximately balanced. Most participants were pursuing their first degree (Undergraduate, 91.8%), while a smaller proportion had previously completed a college-level education before entering university (University, 8.2%). The table also shows differences in personality health (measured by MMPI), psychological flexibility (measured by AAQ and CFQ-F), coping mechanisms (measured by Positive Coping [PC] and Negative Coping [NC] scores), and mental health (measured by the SCL) across gender and first-degree status. Independent-samples t-tests were used to examine these differences. Gender showed significant effects on several variables, indicating it should be treated as a control variable in subsequent analyses. First-degree status did not show significant effects. No significant differences were observed between males and females in terms of social introversion or coping style choices.

**Table 1:** Differences in participant characteristics with independent samples *T*-test.

Variables	Total 1 *N* = 2,528	Female 1 *N* = 1,183	Male 1 *N* = 1,345	*p*-value 2	University 1 *N* = 207	Undergraduate 1 *N* = 2,321	*p*-value 2
Age	20.10 (1.34)	20.15 (1.39)	20.04 (1.29)	0.045	22.56 (0.99)	19.88 (1.13)	<0.001
Time	1,759 (1,182)	1,830 (1,091)	1,697 (1,253)	0.004	1,662 (757)	1,768 (1,212)	0.069
Q	48.5 (4.4)	48.9 (4.8)	48.1 (4.1)	<0.001	48.4 (4.6)	48.5 (4.4)	0.7
L	46 (9)	47 (9)	45 (9)	<0.001	48 (8)	46 (9)	<0.001
F	52 (18)	50 (17)	55 (18)	<0.001	48 (15)	53 (18)	<0.001
K	48 (12)	50 (12)	46 (12)	<0.001	51 (11)	48 (12)	<0.001
Hs	50 (11)	49 (11)	51 (11)	0.004	49 (11)	50 (11)	0.2
D	45 (12)	46 (12)	44 (11)	<0.001	47 (11)	45 (12)	0.047
Hy	47 (10)	48 (10)	47 (11)	<0.001	49 (10)	47 (10)	0.007
Pd	49 (11)	48 (11)	49 (12)	0.018	48 (11)	49 (11)	0.3
Mf	54 (11)	57 (12)	51 (10)	<0.001	58 (11)	53 (11)	<0.001
Pa	50 (13)	49 (12)	52 (14)	<0.001	48 (11)	51 (13)	0.002
Pt	53 (14)	52 (13)	55 (14)	<0.001	51 (12)	54 (14)	<0.001
Sc	53 (15)	50 (14)	55 (16)	<0.001	49 (13)	53 (15)	<0.001
Ma	55 (12)	53 (11)	56 (12)	<0.001	53 (10)	55 (12)	0.002
Si	47 (13)	46 (13)	47 (12)	0.089	47 (13)	47 (12)	>0.9
SOM	1.23 (0.35)	1.26 (0.36)	1.21 (0.34)	<0.001	1.19 (0.29)	1.24 (0.35)	0.039
O-C	1.62 (0.60)	1.68 (0.61)	1.57 (0.57)	<0.001	1.53 (0.54)	1.63 (0.60)	0.016
INT	1.46 (0.53)	1.49 (0.55)	1.42 (0.51)	0.001	1.39 (0.47)	1.46 (0.53)	0.030
DEP	1.36 (0.47)	1.41 (0.49)	1.33 (0.46)	<0.001	1.35 (0.47)	1.37 (0.47)	0.6
ANX	1.33 (0.42)	1.36 (0.44)	1.30 (0.40)	<0.001	1.29 (0.40)	1.33 (0.42)	0.14
HOS	1.31 (0.42)	1.35 (0.44)	1.28 (0.40)	<0.001	1.24 (0.33)	1.32 (0.43)	0.001
PHOB	1.30 (0.43)	1.34 (0.46)	1.26 (0.40)	<0.001	1.24 (0.35)	1.30 (0.43)	0.035
PAR	1.32 (0.42)	1.34 (0.45)	1.29 (0.39)	0.003	1.23 (0.33)	1.32 (0.43)	<0.001
PSY	1.30 (0.40)	1.32 (0.41)	1.29 (0.39)	0.046	1.21 (0.32)	1.31 (0.40)	<0.001
GSI	1.31 (0.41)	1.34 (0.43)	1.29 (0.40)	0.002	1.27 (0.36)	1.32 (0.42)	0.060
T	122 (35)	125 (37)	120 (34)	<0.001	117 (31)	123 (36)	0.014
AT	1.36 (0.39)	1.39 (0.41)	1.33 (0.38)	<0.001	1.30 (0.34)	1.36 (0.40)	0.015
NP	23 (23)	25 (23)	21 (23)	<0.001	20 (20)	23 (23)	0.027
CFQ-F	25 (11)	26 (11)	25 (11)	<0.001	25 (10)	25 (11)	0.5
AAQ	17 (8)	17 (8)	16 (8)	<0.001	16 (7)	17 (8)	0.4
PC	2.01 (0.66)	2.02 (0.63)	2.01 (0.68)	0.5	2.04 (0.59)	2.01 (0.66)	0.5
NC	1.28 (0.64)	1.27 (0.61)	1.30 (0.67)	0.3	1.25 (0.58)	1.29 (0.65)	0.4

1. Average standard deviation (SD).

2. Welch two sample *t*-test.

Table 2 displays the results of one-way ANOVA used to assess the effects of grade and academic subject on the research variables. Both grade and subject significantly influenced several variables and were thus included as control variables. Grade had a broader impact than academic subject. Neither grade nor subject significantly influenced the choice of coping mechanisms. However, psychological flexibility varied significantly across different grades but not across academic subjects. No significant effects were found for age or test timing.

**Table 2:** Differences in participant characteristics with one-way ANOVA.

Variables	Grade 20,201 *N* = 580	Grade 20,211 *N* = 678	Grade 20,221 *N* = 638	Grade 20,231 *N* = 632	*p*-value 2	Physical education 1 *N* = 62	Engineering 1 *N* = 911	Literature 1 *N* = 953	Science 1 *N* = 431	Art 1 *N* = 171	*p*-value 2
Age	22.47 (0.75)	21.33 (0.80)	20.43 (1.05)	19.70 (1.23)	<0.001	20.34 (1.13)	19.93 (1.14)	20.43 (1.54)	19.90 (1.28)	19.51 (0.81)	<0.001
Time	1,599 (1,520)	1,755 (1,318)	1,734 (1,135)	1,774 (1,153)	0.6	1,305 (1,185)	1,731 (1,255)	1,801 (1,146)	1,853 (1,172)	1,605 (929)	0.002
Q	46.8 (3.9)	48.1 (4.4)	48.3 (4.6)	48.6 (4.4)	0.001	47.6 (4.4)	48.3 (4.3)	48.8 (4.6)	48.0 (4.3)	49.4 (4.5)	<0.001
L	47 (10)	47 (9)	47 (9)	46 (9)	0.016	47 (9)	46 (9)	47 (9)	46 (9)	46 (10)	0.080
F	60 (25)	50 (18)	51 (19)	52 (17)	<0.001	59 (20)	54 (19)	51 (17)	51 (16)	55 (19)	<0.001
K	49 (15)	51 (14)	49 (13)	47 (12)	<0.001	47 (11)	47 (13)	49 (12)	48 (11)	47 (12)	0.032
Hs	48 (11)	46 (11)	48 (11)	51 (11)	<0.001	53 (10)	49 (11)	50 (11)	51 (11)	51 (12)	0.006
D	41 (10)	42 (11)	44 (12)	46 (12)	<0.001	44 (10)	44 (11)	46 (12)	46 (12)	45 (12)	<0.001
Hy	45 (9)	45 (9)	45 (10)	48 (11)	<0.001	49 (11)	46 (10)	48 (10)	49 (10)	47 (12)	<0.001
Pd	50 (12)	46 (11)	47 (11)	50 (11)	<0.001	53 (12)	48 (11)	49 (11)	49 (11)	49 (13)	0.039
Mf	51 (10)	52 (12)	53 (11)	54 (11)	0.002	51 (9)	52 (10)	56 (12)	54 (11)	54 (11)	<0.001
Pa	53 (16)	49 (13)	49 (13)	51 (13)	0.004	53 (13)	51 (14)	49 (12)	50 (12)	51 (13)	0.016
Pt	51 (15)	49 (14)	51 (14)	55 (13)	<0.001	54 (12)	54 (14)	53 (14)	54 (13)	54 (14)	0.3
Sc	54 (19)	49 (16)	50 (16)	54 (15)	<0.001	56 (15)	54 (16)	51 (15)	52 (14)	55 (16)	<0.001
Ma	57 (14)	54 (12)	54 (11)	55 (11)	0.020	58 (10)	56 (12)	54 (11)	54 (11)	56 (12)	0.001
Si	44 (11)	44 (13)	45 (13)	48 (12)	<0.001	46 (11)	47 (12)	47 (13)	47 (12)	47 (13)	0.8
SOM	1.12 (0.24)	1.21 (0.35)	1.23 (0.34)	1.25 (0.35)	0.007	1.19 (0.36)	1.22 (0.33)	1.26 (0.37)	1.20 (0.32)	1.29 (0.39)	0.004
O-C	1.27 (0.42)	1.44 (0.50)	1.54 (0.59)	1.69 (0.60)	<0.001	1.43 (0.57)	1.57 (0.57)	1.70 (0.61)	1.57 (0.58)	1.66 (0.61)	<0.001
INT	1.17 (0.36)	1.33 (0.46)	1.39 (0.48)	1.51 (0.55)	<0.001	1.24 (0.41)	1.42 (0.51)	1.52 (0.55)	1.41 (0.51)	1.48 (0.54)	<0.001
DEP	1.15 (0.32)	1.28 (0.40)	1.34 (0.45)	1.39 (0.49)	<0.001	1.19 (0.37)	1.32 (0.44)	1.43 (0.50)	1.33 (0.44)	1.42 (0.50)	<0.001
ANX	1.16 (0.34)	1.25 (0.39)	1.28 (0.37)	1.36 (0.43)	<0.001	1.20 (0.39)	1.29 (0.39)	1.38 (0.45)	1.29 (0.37)	1.38 (0.48)	<0.001
HOS	1.14 (0.29)	1.25 (0.39)	1.30 (0.43)	1.33 (0.43)	<0.001	1.21 (0.33)	1.28 (0.40)	1.35 (0.44)	1.27 (0.41)	1.39 (0.46)	<0.001
PHOB	1.14 (0.36)	1.25 (0.41)	1.26 (0.39)	1.32 (0.44)	<0.001	1.16 (0.35)	1.25 (0.40)	1.35 (0.46)	1.27 (0.41)	1.33 (0.45)	<0.001
PAR	1.13 (0.27)	1.25 (0.39)	1.28 (0.40)	1.35 (0.44)	<0.001	1.16 (0.30)	1.28 (0.38)	1.36 (0.45)	1.30 (0.41)	1.37 (0.53)	<0.001
PSY	1.12 (0.27)	1.24 (0.38)	1.26 (0.37)	1.33 (0.41)	<0.001	1.17 (0.34)	1.28 (0.39)	1.34 (0.42)	1.27 (0.35)	1.33 (0.45)	<0.001
GSI	1.15 (0.28)	1.29 (0.42)	1.29 (0.40)	1.33 (0.42)	<0.001	1.20 (0.39)	1.29 (0.40)	1.35 (0.43)	1.27 (0.37)	1.37 (0.47)	<0.001
T	104 (26)	115 (33)	119 (34)	125 (36)	<0.001	110 (32)	119 (33)	127 (37)	119 (33)	126 (39)	<0.001
AT	1.15 (0.29)	1.28 (0.37)	1.32 (0.38)	1.39 (0.40)	<0.001	1.22 (0.35)	1.32 (0.37)	1.41 (0.41)	1.32 (0.36)	1.40 (0.43)	<0.001
NP	11 (20)	20 (25)	21 (24)	24 (23)	<0.001	14 (22)	21 (23)	25 (23)	21 (23)	25 (24)	<0.001
CFQ-F	19 (10)	22 (9)	24 (10)	27 (11)	<0.001	22 (11)	24 (11)	27 (11)	25 (11)	25 (12)	<0.001
AAQ	13 (6)	15 (7)	16 (7)	17 (8)	<0.001	15 (7)	16 (7)	17 (8)	17 (8)	17 (8)	0.001
PC	2.04 (0.73)	2.04 (0.62)	2.03 (0.67)	2.00 (0.65)	0.7	1.92 (0.72)	2.00 (0.69)	2.04 (0.62)	2.00 (0.66)	2.01 (0.63)	0.6
NC	1.36 (0.75)	1.28 (0.65)	1.31 (0.67)	1.27 (0.63)	0.5	1.34 (0.72)	1.30 (0.68)	1.27 (0.62)	1.26 (0.61)	1.32 (0.60)	0.6

1. Average standard deviation (SD).

2. One-way ANOVA.

Table 3 shows the results of Pearson correlation analysis. Personality health was found to be correlated with mental health, although the strength of this relationship was weak (*r* = 0.042 to 0.14). Psychological flexibility exhibited a moderate and statistically significant correlation with mental health (*r* = 0.454 to 0.660, *p* < 0.001). Coping mechanisms were also significantly correlated with mental health, but the correlation strength was weaker than that of psychological flexibility (*r* = 0.048 to 0.270). Notably, psychological flexibility and coping mechanisms were highly correlated with most personality health scales, except for Masculinity/Femininity (Mf). Psychological flexibility was not significantly associated with Paranoia (Pa) and Hypomania (Ma), while coping mechanisms were not correlated with Hysteria (Hy). The relationship between Depressive personality (D) and negative coping strategies requires further investigation.

**Table 3:** Associations between healthy personality, psychological flexibility, coping mechanisms and mental health among participant.

	Q	L	F	K	Hs	D	Hy	Pd	Mf	Pa	Pt	Sc	Ma	Si	SOM	O-C	INT	DEP	ANX	HOS	PHOB	PAR	PSY	GSI	T	AT	NP	CFQ-F	AAQ-II	PC	NC
L		0.111^***^	0.249^***^	0.127^***^	0.027	0.065^**^	0.105^***^	0.150^***^	0.033	0.208^***^	0.122^***^	0.198^***^	0.243^***^	0.019	0.015	0.028	0.019	0.040^*^	0.018	0.025	0.009	0.012	0.027	0.034	0.024	0.024	0.01	0.026	0.02	0.027	0.003
F			0.477^***^	0.638^***^	0.292^***^	0.012	0.200^***^	0.361^***^	0.074^***^	0.452^***^	0.586^***^	0.590^***^	0.511^***^	0.211^***^	0.034	0.031	0.042^*^	0.016	0.043^*^	0.028	0.033	0.040^*^	0.042^*^	0.033	0.038	0.038	0.052^**^	0.015	0.016	0.032	0.064^**^
K				0.599^***^	0.510^***^	0.105^***^	0.070^***^	0.610^***^	0.216^***^	0.825^***^	0.682^***^	0.867^***^	0.744^***^	0.269^***^	0.062^**^	0.012	0.024	0.014	0.033	0.065^**^	0.026	0.050^*^	0.031	0.022	0.035	0.035	0.045^*^	0.013	0.005	0.075^***^	0.075^***^
Hs					0.427^***^	0.159^***^	0.269^***^	0.376^***^	0.055^**^	0.560^***^	0.806^***^	0.774^***^	0.599^***^	0.449^***^	0.074^***^	0.066^***^	0.075^***^	0.054^**^	0.074^***^	0.073^***^	0.049^*^	0.071^***^	0.073^***^	0.060^**^	0.076^***^	0.076^***^	0.083^***^	0.059^**^	0.047^*^	0.068^***^	0.088^***^
D						0.629^***^	0.585^***^	0.654^***^	0.059^**^	0.558^***^	0.674^***^	0.631^***^	0.345^***^	0.481^***^	0.098^***^	0.108^***^	0.105^***^	0.113^***^	0.109^***^	0.123^***^	0.104^***^	0.099^***^	0.089^***^	0.105^***^	0.121^***^	0.120^***^	0.122^***^	0.086^***^	0.069^***^	0.081^***^	0.053^**^
Hy							0.569^***^	0.453^***^	0.207^***^	0.230^***^	0.471^***^	0.277^***^	0.123^***^	0.628^***^	0.101^***^	0.136^***^	0.130^***^	0.140^***^	0.113^***^	0.120^***^	0.122^***^	0.109^***^	0.100^***^	0.126^***^	0.139^***^	0.139^***^	0.133^***^	0.112^***^	0.102^***^	0.065^**^	0.005
Pd								0.373^***^	0.195^***^	0.070^***^	0.090^***^	0.005	0.196^***^	0.154^***^	0.042^*^	0.074^***^	0.070^***^	0.082^***^	0.061^**^	0.073^***^	0.066^***^	0.058^**^	0.049^*^	0.079^***^	0.075^***^	0.075^***^	0.068^***^	0.060^**^	0.054^**^	0.025	0.031
Mf									0.006	0.642^***^	0.619^***^	0.641^***^	0.463^***^	0.339^***^	0.087^***^	0.092^***^	0.112^***^	0.087^***^	0.095^***^	0.099^***^	0.076^***^	0.103^***^	0.097^***^	0.090^***^	0.107^***^	0.107^***^	0.104^***^	0.062^**^	0.056^**^	0.080^***^	0.064^**^
Pa										0.039^*^	0	0.123^***^	0.186^***^	0.084^***^	0.029	0.058^**^	0.036	0.041^*^	0.033	0.024	0.060^**^	0.012	0.026	0.026	0.042^*^	0.042^*^	0.049^*^	0.049^*^	0.048^*^	0.012	0.027
Pt											0.716^***^	0.819^***^	0.658^***^	0.278^***^	0.069^***^	0.035	0.047^*^	0.038	0.052^**^	0.065^**^	0.032	0.074^***^	0.051^*^	0.038	0.055^**^	0.055^**^	0.064^**^	0.005	0.014	0.078^***^	0.061^**^
Sc												0.893^***^	0.598^***^	0.598^***^	0.117^***^	0.122^***^	0.122^***^	0.102^***^	0.125^***^	0.123^***^	0.107^***^	0.117^***^	0.111^***^	0.108^***^	0.131^***^	0.131^***^	0.137^***^	0.087^***^	0.075^***^	0.102^***^	0.082^***^
Ma													0.752^***^	0.453^***^	0.100^***^	0.081^***^	0.080^***^	0.063^**^	0.088^***^	0.102^***^	0.068^***^	0.095^***^	0.084^***^	0.075^***^	0.093^***^	0.093^***^	0.101^***^	0.045^*^	0.047^*^	0.099^***^	0.087^***^
Si														0.026	0.061^**^	0.024	0.022	0.01	0.048^*^	0.049^*^	0.023	0.045^*^	0.044^*^	0.026	0.037	0.038	0.050^*^	0.011	0.008	0.043^*^	0.083^***^
SOM															0.096^***^	0.131^***^	0.137^***^	0.120^***^	0.109^***^	0.115^***^	0.130^***^	0.106^***^	0.096^***^	0.105^***^	0.132^***^	0.132^***^	0.132^***^	0.099^***^	0.101^***^	0.108^***^	0.044^*^
O-C																0.653^***^	0.643^***^	0.682^***^	0.767^***^	0.669^***^	0.621^***^	0.662^***^	0.688^***^	0.716^***^	0.811^***^	0.811^***^	0.817^***^	0.454^***^	0.494^***^	0.223^***^	0.075^***^
INT																	0.826^***^	0.800^***^	0.785^***^	0.677^***^	0.688^***^	0.722^***^	0.756^***^	0.706^***^	0.893^***^	0.893^***^	0.817^***^	0.645^***^	0.597^***^	0.209^***^	0.048^*^
DEP																		0.847^***^	0.812^***^	0.702^***^	0.735^***^	0.785^***^	0.802^***^	0.707^***^	0.913^***^	0.913^***^	0.845^***^	0.623^***^	0.628^***^	0.241^***^	0.070^***^
ANX																			0.832^***^	0.718^***^	0.720^***^	0.754^***^	0.812^***^	0.749^***^	0.924^***^	0.924^***^	0.854^***^	0.588^***^	0.621^***^	0.259^***^	0.088^***^
HOS																				0.723^***^	0.740^***^	0.768^***^	0.820^***^	0.761^***^	0.923^***^	0.923^***^	0.879^***^	0.595^***^	0.615^***^	0.234^***^	0.057^**^
PHOB																					0.606^***^	0.735^***^	0.703^***^	0.684^***^	0.811^***^	0.812^***^	0.785^***^	0.499^***^	0.536^***^	0.244^***^	0.095^***^
PAR																						0.645^***^	0.672^***^	0.613^***^	0.804^***^	0.804^***^	0.779^***^	0.488^***^	0.512^***^	0.205^***^	0.064^**^
PSY																							0.804^***^	0.709^***^	0.858^***^	0.858^***^	0.823^***^	0.532^***^	0.556^***^	0.240^***^	0.063^**^
GSI																								0.747^***^	0.896^***^	0.896^***^	0.850^***^	0.568^***^	0.594^***^	0.239^***^	0.074^***^
T																									0.840^***^	0.840^***^	0.810^***^	0.522^***^	0.548^***^	0.227^***^	0.058^**^
AT																										1.000^***^	0.947^***^	0.643^***^	0.660^***^	0.265^***^	0.078^***^
NP																											0.947^***^	0.643^***^	0.660^***^	0.265^***^	0.078^***^
CFQ-F																												0.582^***^	0.618^***^	0.270^***^	0.061^**^
AAQ																													0.811^***^	0.185^***^	0.028
PC																														0.251^***^	0.051^*^
NC																															0.306^***^

***p < 0.001.

**Figure 2:**
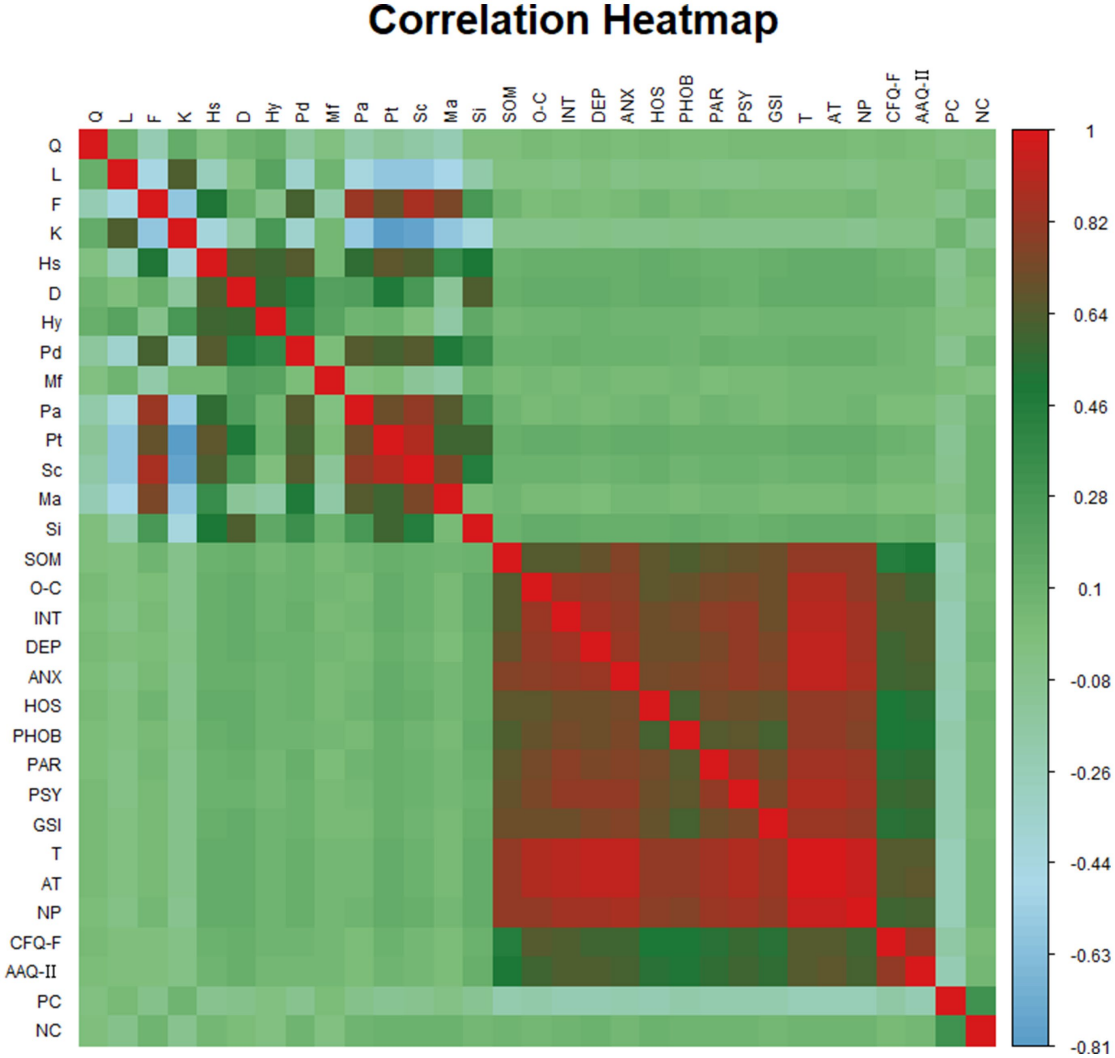
Summarizes the results of correlation analysis.

Figure 2 provides a heatmap summarizing the correlation analysis. The color intensity reflects the strength of the correlations, with darker colors indicating stronger associations. The heatmap is divided into four quadrants. The lower left and upper right quadrants suggest direct correlations between personality health and mental health, although the correlations are relatively weak, suggesting the presence of mediating or moderating variables. The upper left quadrant, which represents inter-correlations among personality health factors, shows more complex and less cohesive patterns. In contrast, the lower right quadrant, representing correlations among mental health factors, shows stronger and more consistent relationships, indicating that mental health variables form a more unified construct. Additionally, psychological flexibility and coping mechanisms show strong associations with both personality health and mental health variables, with psychological flexibility demonstrating stronger connections.

Table 4 summarizes the results of hierarchical multiple regression models predicting mental health outcomes. A stepwise modeling approach was used. Model 1 included only personality health variables, yielding a weak explanatory capacity. Model 2 added psychological flexibility, significantly increasing the model’s predictive power. Model 3 included coping mechanisms, and Model 4 combined all three categories. The inclusion of psychological flexibility and coping mechanisms significantly improved the explanatory power of the models. Model 4 reached a moderate level of predictive accuracy (Adjusted *R*^2^ > 0.30). These findings suggest that while personality health is related to mental health, its effects are more indirect and are better explained through intermediate variables such as psychological flexibility and coping mechanisms.

**Table 4:** Regression model between healthy personality, psychological flexibility, coping mechanisms and mental health among participant.

Characteristic	Model 1			Model 2			Model 3			Model 4		
	Beta^1^	SE^2^	95%CI^2^	Beta^1^	SE^2^	95%CI^2^	Beta^1^	SE^2^	95%CI^2^	Beta^1^	SE^2^	95%CI^2^
Q	0.22	0.165	−0.11 0.54	0.08	0.158	−0.23 0.39	0.06	0.120	−0.17, 0.30			
L	0.10	0.106	−0.11, 0.30	0.05	0.101	−0.15, 0.25	0.01	0.077	−0.14, 0.10			
F	−0.21**	0.100	−0.41, −0.02	−0.19**	0.095	−0.38, 0.00	−0.04	0.072	−0.18, 0.10			
K	0.11	0.127	−0.14, 0.36	0.08	0.121	−0.15, 0.32	0.13	−0.23	−0.05, 0.31			
Hs	0.04	0.137	−0.23, 0.31	0.01	0.131	−0.24, 0.27	0.02	−0.17	−0.17, 0.22			
D	0.11	0.111	−0.11, 0.33	0.17	0.106	−0.04, 0.38	0.05	0.08	−0.11, 0.21			
Hy	−0.03	0.131	−0.29, 0.23	0.01	0.125	−0.24, 0.26	−0.02	0.095	−0.21, 0.16			
Pd	0.19*	0.099	0.01,0.38	0.12	0.095	−0.06, 0.31	0.07	0.072	−0.07, 0.21			
Mf	0.06	0.068	−0.07, 0.20	0.08	0.065	−0.05, 0.20	0.02	0.050	−0.08, 0.11			
Pa	−0.21	0.107	−0.33, 0.09	−0.12	0.102	−0.32, 0.08	−0.03	0.077	−0.18, 0.12			
Pt	0.27	0.169	−0.06, 0.60	0.23	0.161	−0.08, 0.55	0.21*	−0.123	0.03, 0.45			
Sc	0.13	0.175	−0.21, 0.48	0.06	0.167	−0.27, 0.39	0.00	0.127	−0.25, 0.25			
Ma	0.18	0.118	−0.05, 0.41	0.18	0.113	−0.04, 0.40	0.03	0.086	−0.13, 0.20			
Si	0.16*	0.089	0.01, 0.34	0.07	0.085	−0.09, 0.24	0.02	0.065	−0.11, 0.15			
Pc				9.2***	1.09	7.0, 11	4.9***	0.836	3.3, 3.6	5.2***	0.829	3.6, 6.9
Nc				−16***	1.07	−19, −14	−7.4	0.836	−9.0, 5.7	−7.8***	0.838	−9.4, −6.2
CFQ-F							1.0***	0.078	0.86, 1.2	1.0 ***	0.078	0.89, 1.2
AAQ-II							1.6***	0.115	1.4, 1.9	1.6 ***	0.114	1.4, 1.9
*R* ^2^	0.033			0.119			0.495			0.489		
Adjusted *R*^2^	0.027			0.114			0.491			0.489		
*p*-value	<0.001			<0.001			<0.001			<0.001		
Number of Observations	2.528			2.528			2.528			2.528		

1 *p < 0.1; **p < 0.05; **p < 0.01.

2 SE, standard error; Cl, confidence interval.

## 5 Discussion

### 5.1 Core findings and theoretical implications

This study examined the relationships between personality health, psychological flexibility, coping mechanisms, and mental health in Chinese university students. The results revealed significant correlations, with psychological flexibility showing the strongest association. These findings suggest that psychological symptoms arise not only from stable personality traits but also from dynamic processes such as flexibility and coping strategies. Unlike personality traits, psychological flexibility and coping mechanisms can be more readily modified, offering practical intervention opportunities.

Our findings align with prior research (Yang et al., 2019; Hayes et al., 2023), showing a decline in university student mental health post-COVID-19. Male students reported better psychological flexibility and mental health than females, consistent with previous studies (Nolen-Hoeksema, 2003). Additionally, psychological flexibility and mental health were influenced by gender and academic grade but not by age, highlighting the need for targeted interventions.

This study also emphasizes the need for better mental health education, as personality health issues were prevalent. The high abnormal detection rates suggest that updates to the MMPI scale are necessary for cultural relevance in China. Coping mechanisms did not vary significantly, indicating that both positive and negative strategies are used based on individual differences.

### 5.2 Practical applications and interventions

The study highlights the significant role of psychological flexibility and coping mechanisms in mental health, particularly anxiety and depression, which are strongly associated with these factors (Chen et al., 2022). Enhancing psychological flexibility provides a quick intervention, while addressing maladaptive coping strategies can significantly reduce mental health issues. The positive correlation between psychological flexibility and mental health supports interventions like Acceptance and Commitment Therapy (ACT), which has been effective in improving mental health (Peltz et al., 2020; Wang, 2015; Grégoire et al., 2018).

Given cultural differences, interventions should integrate ACT with Chinese practices such as mindfulness and meditation to increase engagement. University counselors should be trained to understand and address the unique stressors faced by students, offering culturally sensitive and personalized support.

### 5.3 Limitations and future directions

Self-report measures may introduce bias, and future research should incorporate objective measures or longitudinal data to provide a more comprehensive understanding. The lack of interaction effects warrants further exploration of how personality traits, coping styles, and psychological flexibility interact to influence mental health. Future studies could use advanced statistical techniques to uncover deeper relationships between these variables.

In conclusion, this study underscores the importance of psychological flexibility and coping mechanisms in mental health and suggests that targeted interventions can improve student well-being. Future research should explore causal relationships and the effectiveness of interventions through longitudinal studies and comprehensive measures of psychological flexibility.

## Data Availability

The original contributions presented in the study are included in the article/supplementary material, further inquiries can be directed to the corresponding author.
