# Correction: The role of healthy personality, psychological flexibility, and coping mechanisms in university students' mental health in China

**DOI:** 10.3389/fpsyg.2025.1680440

**Published:** 2026-01-29

**Authors:** Xiao Yang, Kartini Ilias, Khairil Anuar Md Isa, Qing-hong Li, Hai-bin Wang, Huan Li

**Affiliations:** 1 School of Educational Sciences, Huangshan University, ShuaiShui Campus, Huangshan, Anhui, China; 2 Faculty of Health Sciences, Universiti Teknologi MARA, UiTM Puncak Alam Campus, Puncak Alam, Selangor, Malaysia; 3 Business and Consumer Health Sciences (BiZ-HeALtH), Universiti Teknologi MARA, Cawangan Selangor, Puncak Alam Campus, Selangor, Malaysia; 4 School of Mathematics and Statistics, Huangshan University, ShuaiShui Campus, Huangshan, Anhui, China; 5 School of Economics and Management, Huangshan University, ShuaiShui Campus, Huangshan, Anhui, China

**Keywords:** personality health, psychological flexibility, coping mechanisms, mental health, university students

There was a mistake in Table 4 as published. The regression analysis contains incomplete data, numerous blank cells, and confidence intervals that are shown as single values instead of proper ranges. In addition, several numeric values have been misaligned (e.g., lower-row values appearing in upper rows), and the adjusted *R*-squared value is negative after model adjustment.

**Table:** 

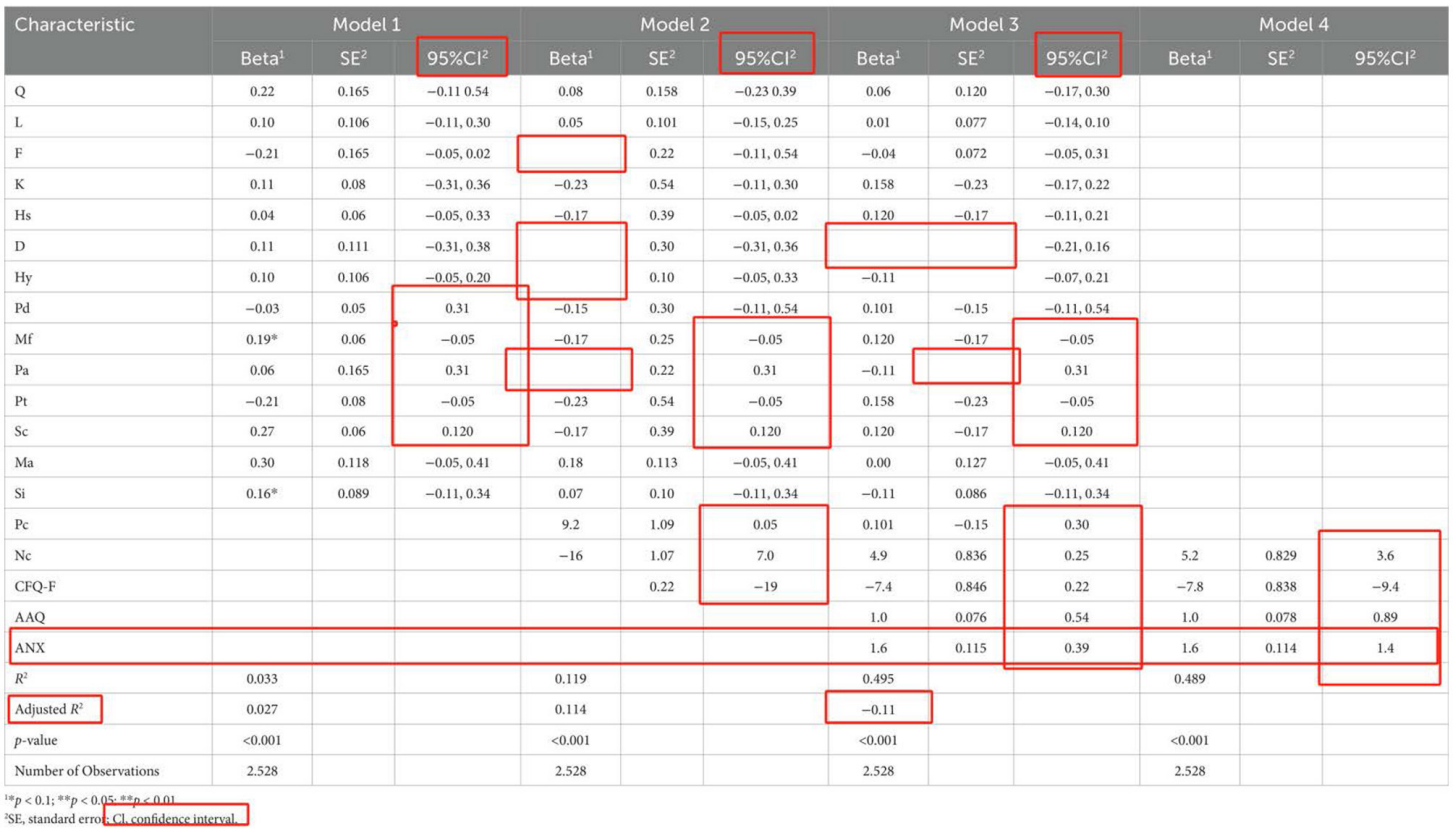

The corrected Table 4 appears below

**Table 4:** Regression model between healthy personality, psychological flexibility, coping mechanisms and mental health among participant.

**Characteristic**	**Model 1**			**Model 2**			**Model 3**			**Model 4**		
	**Beta**	**SE**	**95% CI**	**Beta**	**SE**	**95% CI**	**Beta**	**SE**	**95% CI**	**Beta**	**SE**	**95% CI**
Q	0.22	0.165	−0.11 0.54	0.08	0.158	−0.23 0.39	0.06	0.120	−0.17, 0.30			
L	0.10	0.106	−0.11, 0.30	0.05	0.101	−0.15, 0.25	0.01	0.077	−0.14, 0.16			
F	−0.21^**^	0.100	−0.41, −0.02	−0.19^**^	0.095	−0.38, 0.00	−0.04	0.072	−0.18, 0.10			
K	0.11	0.127	−0.14, 0.36	0.08	0.121	−0.15, 0.32	0.13	−0.23	−0.05, 0.31			
Hs	0.04	0.137	−0.23, 0.31	0.01	0.131	−0.24, 0.27	0.02	−0.17	−0.17, 0.22			
D	0.11	0.111	−0.11, 0.33	0.17	0.106	−0.04, 0.38	0.05	0.08	−0.11,0.21			
Hy	−0.03	0.131	−0.29, 0.23	0.01	0.125	−0.24, 0.26	−0.02	0.095	−0.21, 0.16			
Pd	0.19^*^	0.099	0.01, 0.38	0.12	0.095	−0.06, 0.31	0.07	0.072	−0.07, 0.21			
Mf	0.06	0.068	−0.07, 0.20	0.08	0.065	−0.05, 0.20	0.02	0.050	−0.08, 0.11			
Pa	−0.12	0.107	−0.33, 0.09	−0.12	0.102	−0.32, 0.08	−0.03	0.077	−0.18, 0.12			
Pt	0.27	0.169	−0.06, 0.60	0.23	0.161	−0.08, 0.55	0.21^*^	−0.123	0.03, 0.45			
Sc	0.13	0.175	−0.21, 0.48	0.06	0.167	−0.27, 0.39	0.00	0.127	−0.25, 0.25			
Ma	0.18	0.118	−0.05, 0.41	0.18	0.113	−0.04, 0.40	0.03	0.086	−0.13, 0.20			
Si	0.16^*^	0.089	0.01, 0.34	0.07	0.085	−0.09, 0.24	0.02	0.065	−0.11, 0.15			
Pc				9.2^***^	1.09	7.0, 11	4.9^***^	0.836	3.3, 3.6	5.2^***^	0.829	3.6, 6.9
Nc				−16^***^	1.07	−19.−14	−7.4^***^	0.846	−9.0, −5.7	−7.8^***^	0.838	−9.4, −6.2
CFQ-F							1.0^***^	0.078	0.86, 1.2	1.0^***^	0.078	0.89, 1.2
AAQ-II							1.6^***^	0.115	1.4, 1.9	1.6^***^	0.114	1.4, 1.9
*R*2	0.033			0.119			0.495			0.489		
Adjusted *R*2	0.027			0.114			0.491			0.489		
*p*-value	< 0.001			< 0.001			< 0.001			< 0.001		
Number of observations	2.528			2.528			2.528			2.528		

^*^p < 0.1; ^**^p < 0.05; ^***^p < 0.01. SE, standard error; CI, confidence interval.

The original version of this article has been updated.

